# Supplementary material for: Evidence of direct and indirect reciprocity in network-structured economic games
Source: Commun Psychol. 2024 May 22;2:44. doi: 10.1038/s44271-024-00098-1 (PMC11332088; doi:10.1038/s44271-024-00098-1)

Supplementary Materials for: Evidence of direct and indirect reciprocity in network-structured economic games

Daniel Redhead[1,2,3*†], Matthew Gervais[4], Kotrina Kajokaite[1], Jeremy Koster[1], Arlenys Hurtado Manyoma[1], Danier Hurtado Manyoma[1], Richard McElreath[1] and Cody T. Ross[1*†]

[1]Department of Human Behavior, Ecology and Culture, Max Planck Institute for Evolutionary Anthropology, Leipzig,Germany.
[2]Department of Sociology, University of Groningen, Groningen, The Netherlands.
[3]Inter-University Center for Social Science Theory and Methodology (ICS), University of Groningen, Groningen, The Netherlands.
[4]Division of Psychology, Department of Life Science, Brunel University, London, United Kingdom.

*Corresponding author(s). E-mail(s): daniel_redhead@eva.mpg.de; cody_ross@eva.mpg.de;
†These authors contributed equally.

Fig. S1: Digraphs of two dyadic peer-rating networks and three network-structured economic game networks from the coastal community in rural Colombia. Each network layer is dense, impeding visual assessments of network structure. To gain a better understanding of how perceptions of generosity and selfishness structure behavior in the game networks, we use hive plots in the main manuscript.

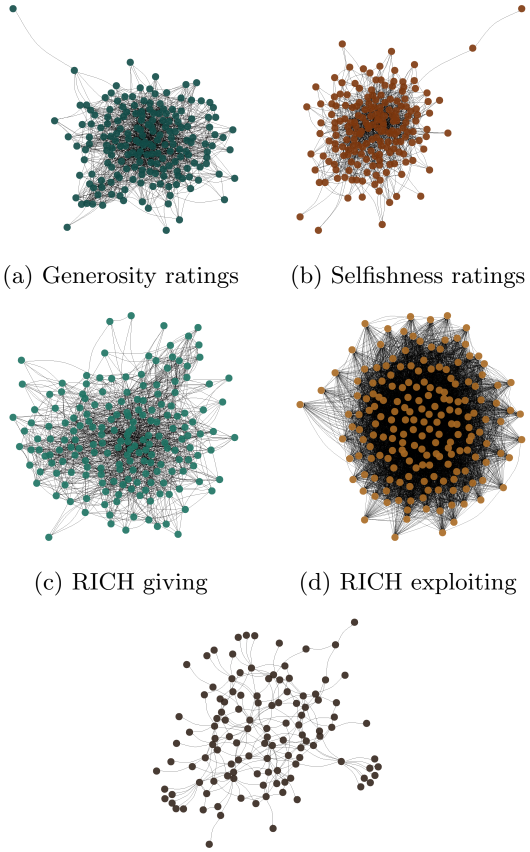

(a) Generosity ratings

(b) Selfishness ratings

(c) RICH giving

(d) RICH exploiting

(e) RICH punishment

Fig. S2: Digraphs of two dyadic peer-rating networks and three network-structured economic game networks from the lowland community in rural Colombia. Each network layer is dense, impeding visual assessments of network structure. To gain a better understanding of how perceptions of generosity and selfishness structure behavior in the game networks, we use hive plots in the main manuscript.

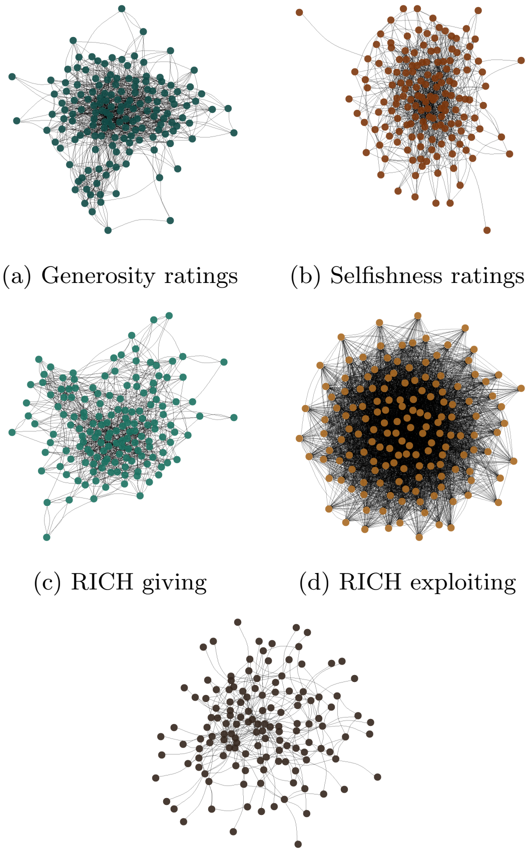

(a) Generosity ratings

(b) Selfishness ratings

(c) RICH giving

(d) RICH exploiting

(e) RICH punishment

Fig. S3: Digraphs of two dyadic peer-rating networks and three network-structured economic game networks from the highland community in rural Colombia. Each network layer is dense, impeding visual assessments of network structure. To gain a better understanding of how perceptions of generosity and selfishness structure behavior in the game networks, we use hive plots in the main manuscript.

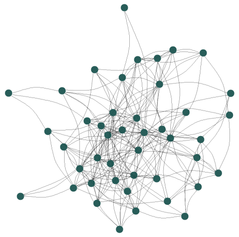

(a) Generosity ratings

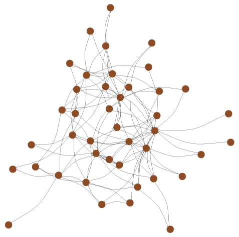

(b) Selfishness ratings

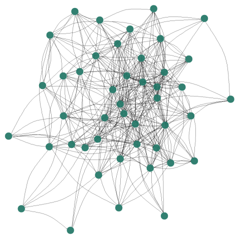

(c) RICH giving

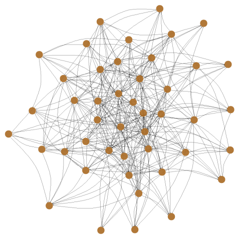

(d) RICH exploiting

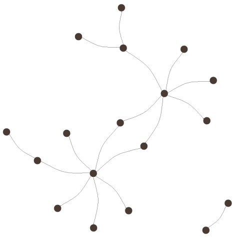

(e) RICH punishment

Fig. S4: Digraphs of two dyadic peer-rating networks and three network-structured economic game networks from the altiplano community in rural Colombia. Each network layer is dense, impeding visual assessments of network structure. To gain a better understanding of how perceptions of generosity and selfishness structure behavior in the game networks, we use hive plots in the main manuscript.

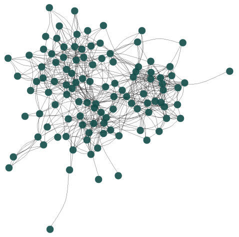

(a) Generosity ratings

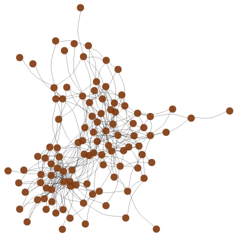

(b) Selfishness ratings

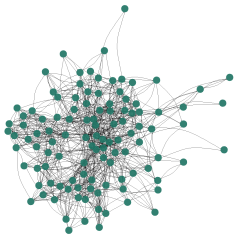

(c) RICH giving

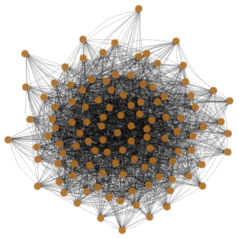

(d) RICH exploiting

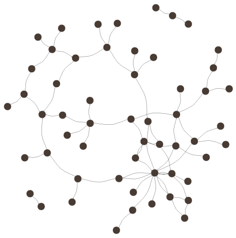

(e) RICH punishment

Fig. S5: Dyad-level correlations in random effects at the coastal site. We plot posterior mean values of all dyadic correlation parameters organized in matrix form.

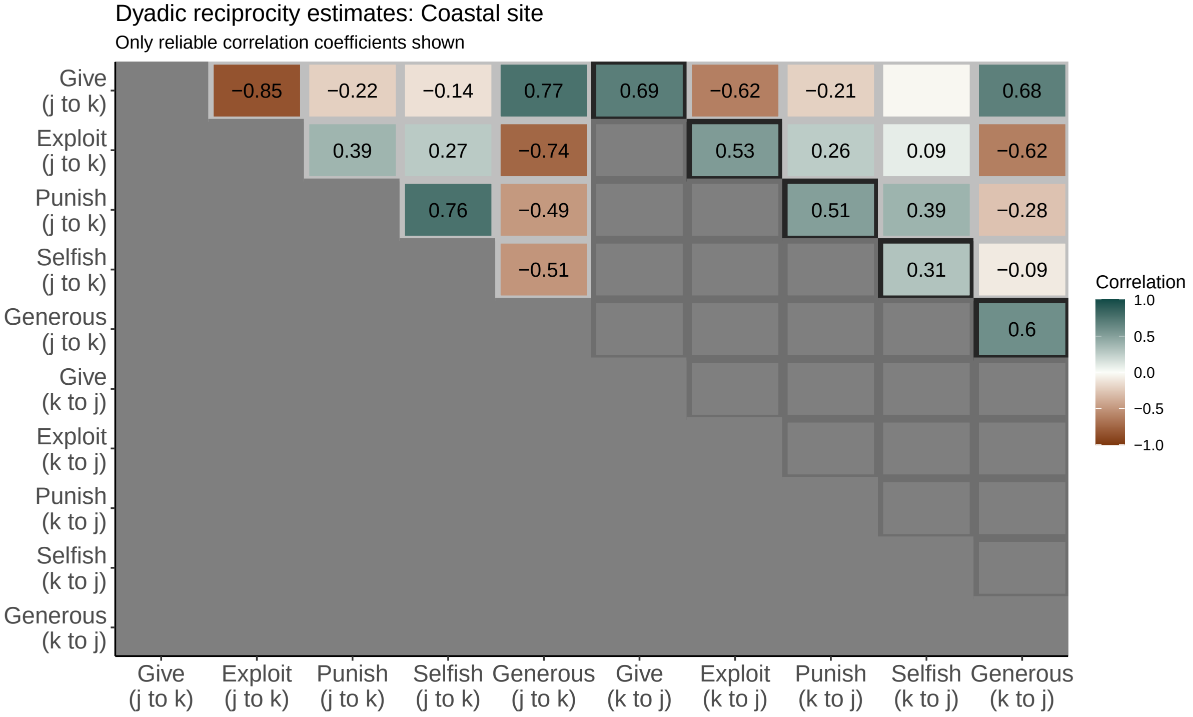

Fig. S6: Generalized correlations in individual-level sender and receiver random effects at the coastal site. We plot posterior mean values of all generalized reciprocity correlation parameters organized in matrix form.

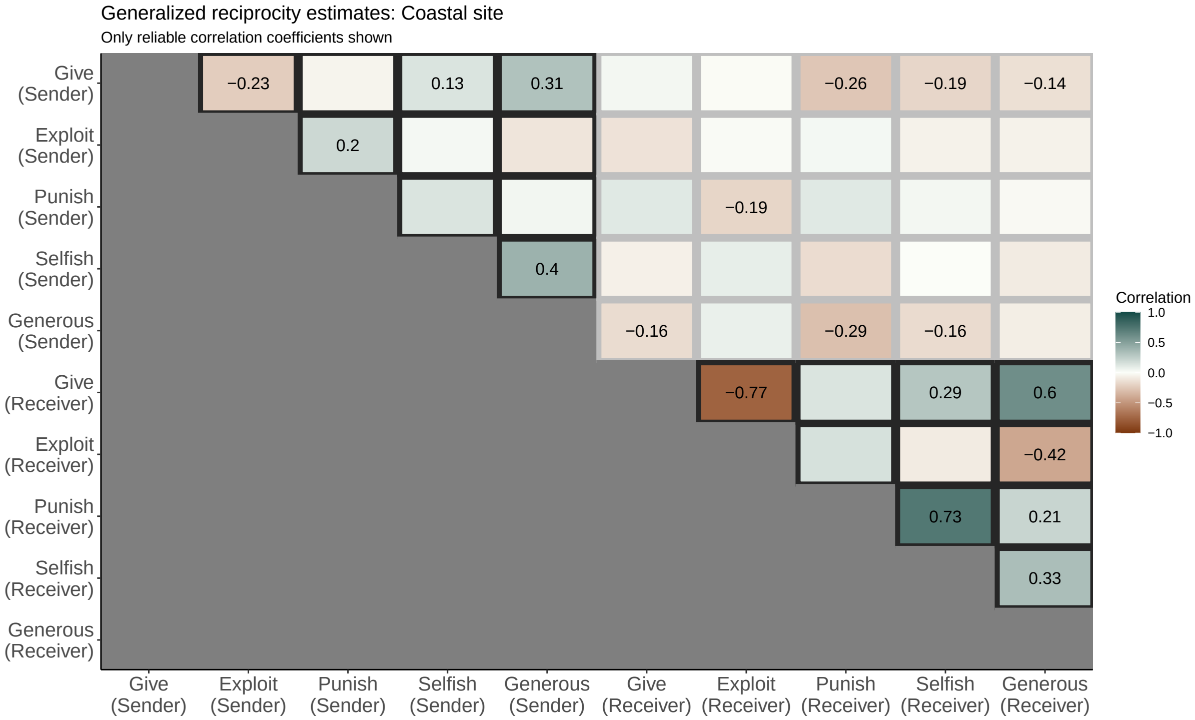

Fig. S7: Dyad-level correlations in random effects at the lowland site. We plot posterior mean values of all dyadic correlation parameters organized in matrix form.

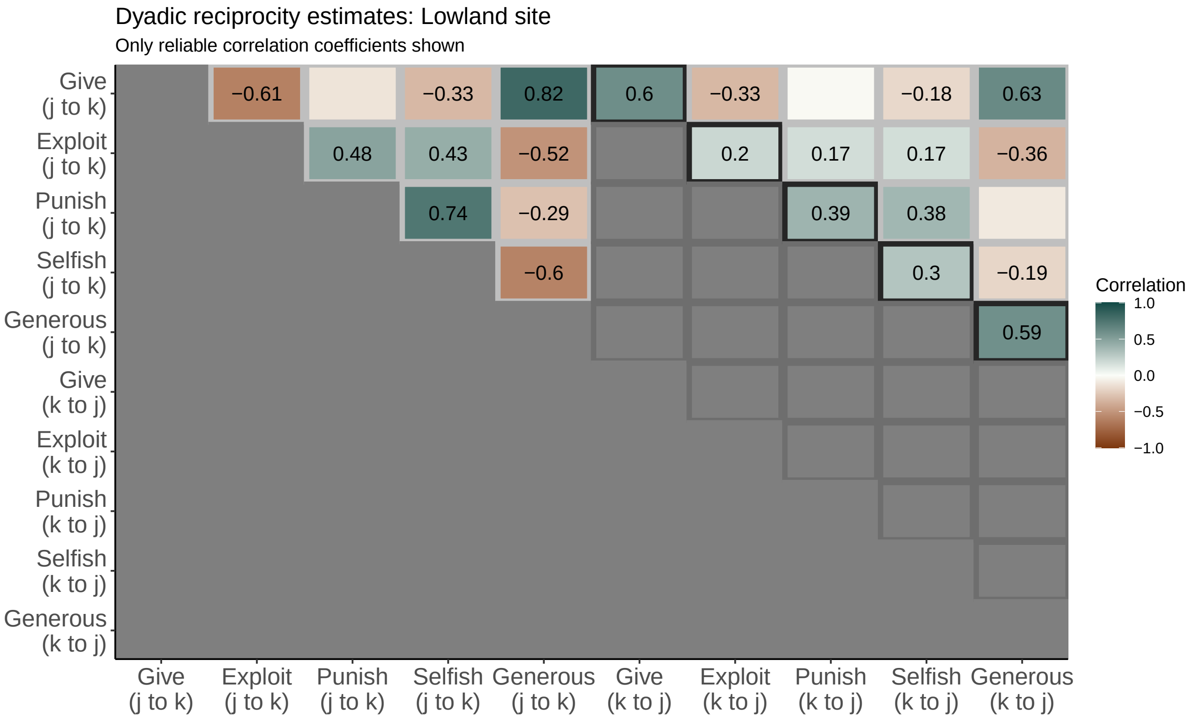

Fig. S8: Generalized correlations in individual-level sender and receiver random effects at the lowland site. We plot posterior mean values of all generalized reciprocity correlation parameters organized in matrix form.

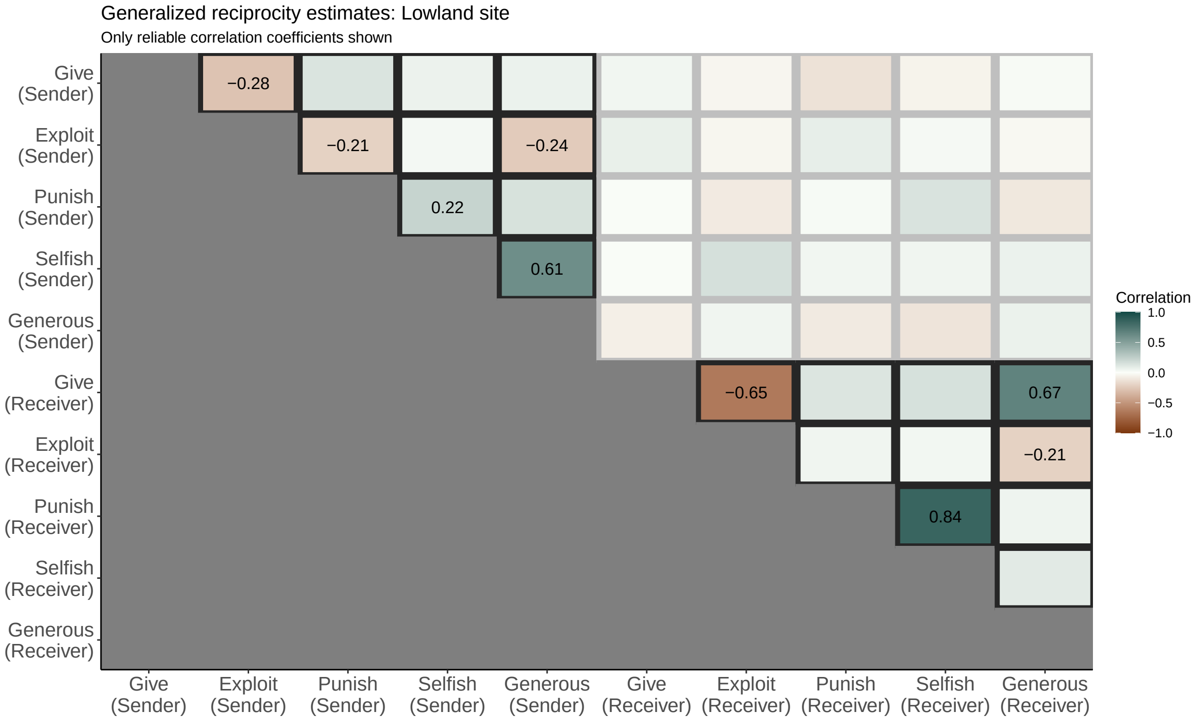

Fig. S9: Dyad-level correlations in random effects at the highland site. We plot posterior mean values of all dyadic correlation parameters organized in matrix form.

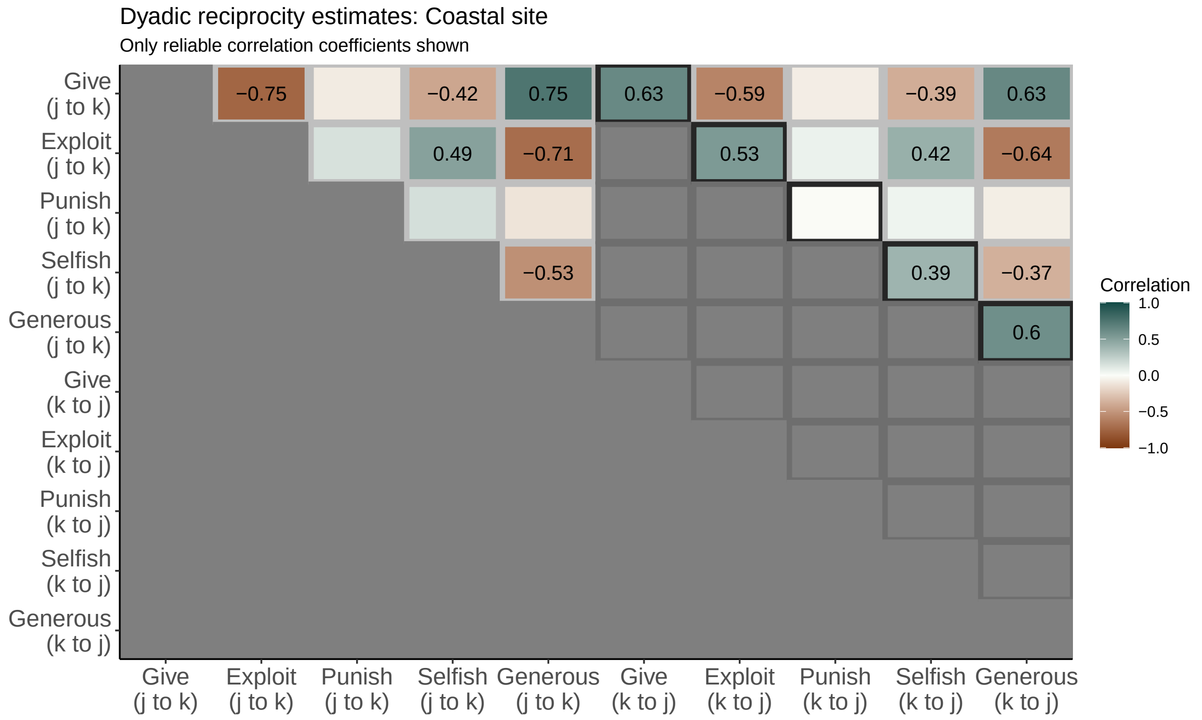

Fig. S10: Generalized correlations in individual-level sender and receiver random effects at the highland site. We plot posterior mean values of all generalized reciprocity correlation parameters organized in matrix form.

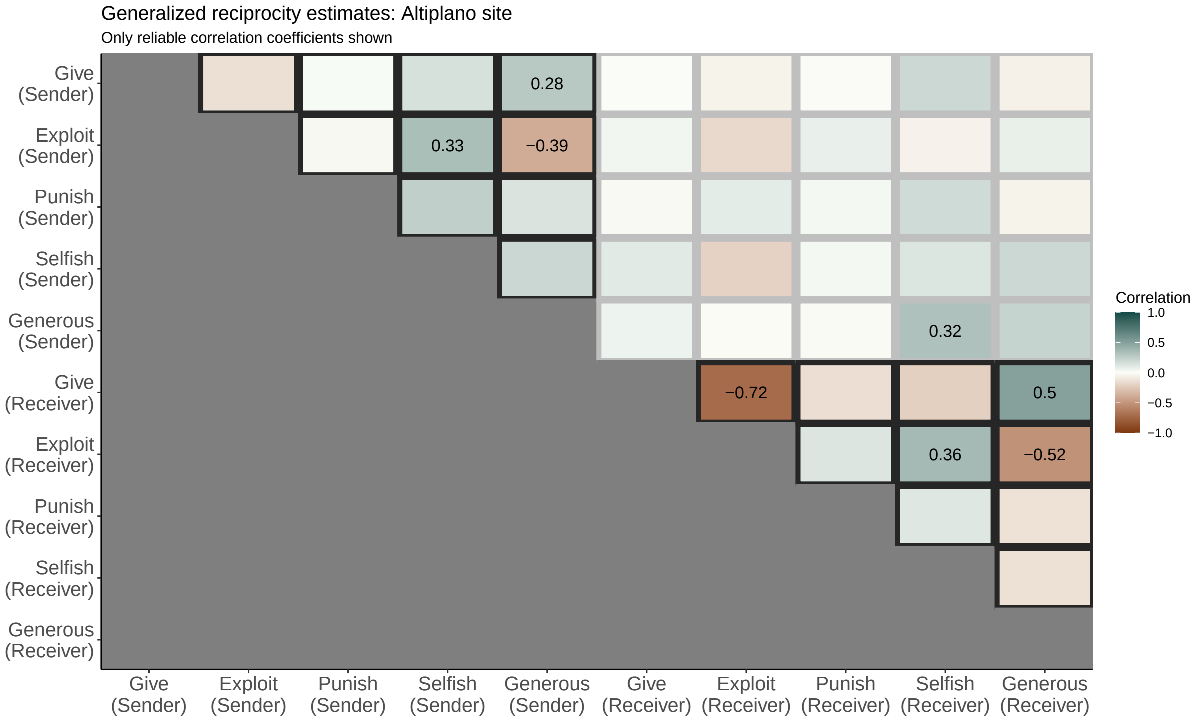

Fig. S11: Dyad-level correlations in random effects at the altiplano site. We plot posterior mean values of all dyadic correlation parameters organized in matrix form.

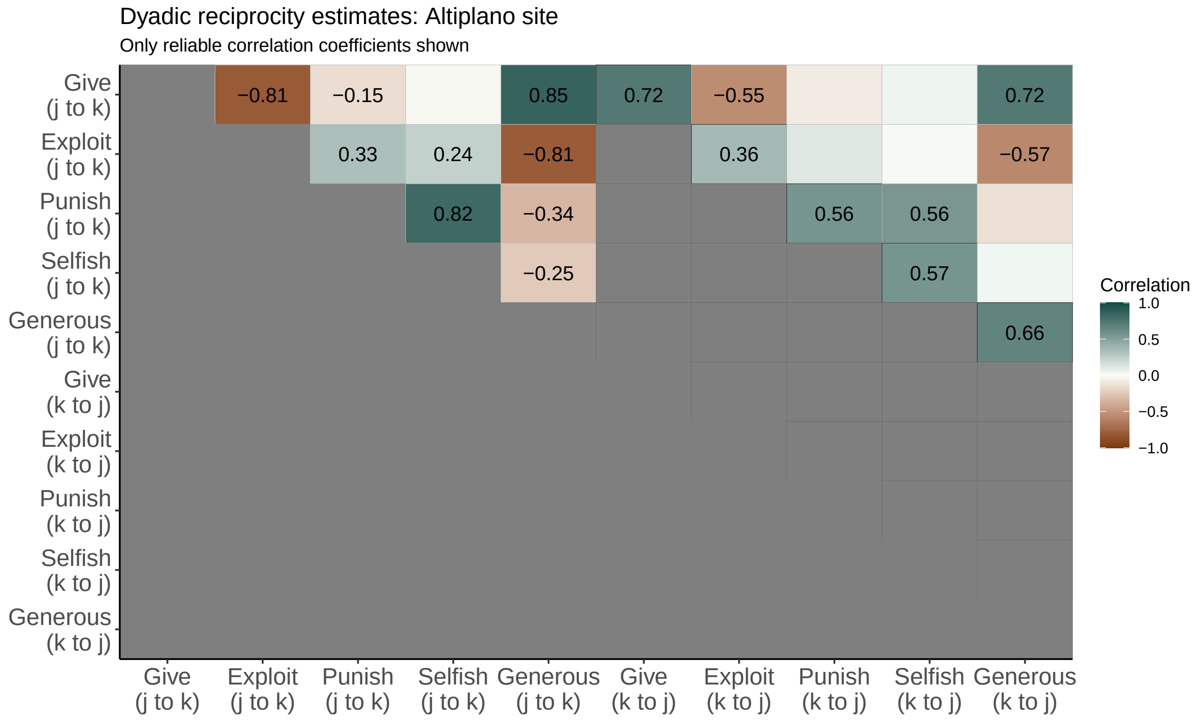

Fig. S12: Generalized correlations in individual-level sender and receiver random effects at the altiplano site. We plot posterior mean values of all generalized reciprocity correlation parameters organized in matrix form.

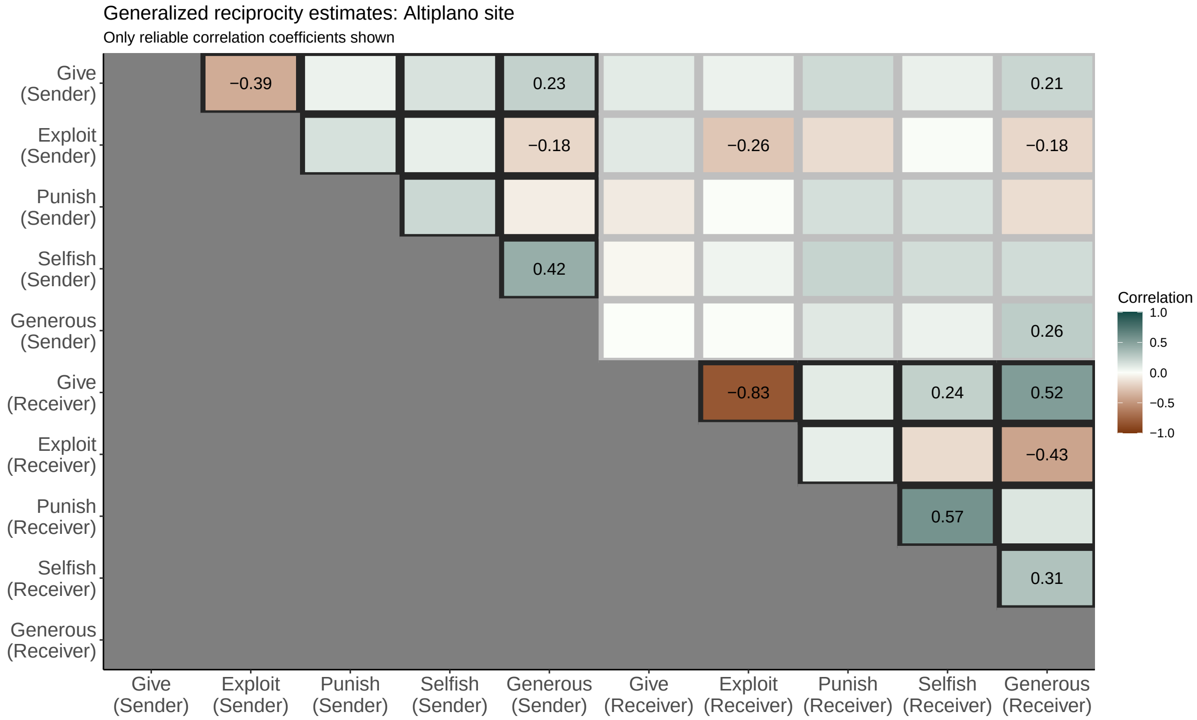

Supplement: Supplementary file 2 — Supplementary Information [file 44271_2024_98_MOESM2_ESM.pdf]
